# Mr. Whyte, on Vaccinia

**Published:** 1801-03

**Authors:** D. Whyte

**Affiliations:** Pera of Constantinople


### *To the Editors of the Medical and Phyſical Journal.*

Gentlemen,

It muſt prove ſatisfactory to the friends of humanity, to hear of the extenſion of the Vaccine inoculation to this diſtant quarter: But I am ſorry to add, that it is not likely ſoon to be generally adopted, and that fears are entertained of its falling into diſuſe the moment I depart.

Although at the head of thoſe I have inoculated, ſtands the ſon and heir of his Majeſty's Ambaſſador Extraordinary; although he and four others, on whom the inoculation has ſucceeded, have gone through the different ſtages of the diſeaſe in the mildeſt manner poſſible; and although the natural ſmall-pox is at preſent committing mighty ravages in a neighbouring village, ſtill no impreſſion can be made on the ſtupid and torpid race of men who conſtitute the motley population of this capital. Abandoned to indolence, their whole life is little better than a vegetable exiſtence; and they are neither to be rouſed from their lethargy by the force of reaſon, nor the fear of danger.

But perhaps I am ill adapted for a medical miſſionary; I can neither court, nor fawn, nor flatter; nor can I bring myſelf to reliſh the greater number of Greek, or even Frank ſocieties, where the converſation is, for the moſt part, as inſipid as the prevailing manners are mean and loathſome.

To make amends for the uncourtly reception the *vaccinia* has experienced in this place, I have lately tranſmitted, by an overland conveyance, a piece of rag, impregnated therewith, to one of my friends at Bombay; and a ſimilar one to Mr. Werry, Engliſh Conſul at Smyrna, who propoſes immediately inoculating one of his own children.

The firſt matter with which I attempted to inoculate Lord Bruce, was contained on a thread which I applied to a ſmall ſcratch made with the point of a lancet; but no inflammation of any kind enſued. After an interval of eight days, the operation was repeated on both arms; but ſtill the attempt failed. At laſt, we received matter in conſiderable quantity, on a rag, with which charging a lancet, I was enabled to perform ſucceſsful inoculation. The failure of the two firſt attempts was leſs owing, I apprehend, to the nature of the virus contained on the thread, which was no older than that which afterwards ſucceeded, and was collected and forwarded from Vienna by the ſame reſpectable gentleman, Dr. Del Carro, than to the

 difficulty difficulty of applying and retaining a fmall and fine bit of thread over a very flight fcratch.

Reflecting on what had happened, I propofed that the third experiment fhould be made by previoufly applying a veficatory of the fize of a fmall pea, and on dividing the raifed cuticle, applying to the naked fibre a fmall morfel of impregnated rag; "for it is poffible," faid I, "that by the matter adhering too firmly, or too flenderly, to the point of the lancet, it may be either detained behind, at the lip of the orifice, or the requifite feparation not enfue." But parental fondnefs over-ruled my reafoning, and I was obliged to perform the operation in the ufual way. I endeavoured, however, by introducing the lancet obliquely, retaining it a few feconds in the wound, and preffing on the part with my thumb as I withdrew it, to infure fuccefs. Succefs followed, but I did not conceive myfelf warranted to difmifs my fears; and accordingly, in the inoculation of two other children, I employed on one arm the mode by incifion, and on the other, that by vefication. In both ways the difeafe was communicated; but with this difference, that the local inflammation took place in the veficated arm on the fourth day, while the incifed one fuffered no change till the ninth. As was predicted, from a knowledge of the general law of the difeafe, there was only one conftitutional affection, and in both cafes it accompanied the inflammation of the veficated arm. Of five other children whom I inoculated at nearly the fame time, by incifion, only one received the difeafe. One of the remaining four I have fince fuccefsfully inoculated by vefication; but to a fecond attempt, in either way, the parents of the others will not at prefent fubmit.

My practice in this difeafe has at no time been extenfive; but my obfervation has not been, on that account, lefs accurate, and I feel no hefitation in recommending inoculation by a veficatory, in preference to that by incifion; the one being often liable to fail, while the other is in its nature almoft infallible. In the former manner, I have been fince informed, the Greeks fometimes inoculate for the fmall-pox. The principal objection that can be made againft it, is the fear of its being attended with greater pain to the patient; but if an infant's pain is to be meafured by its cries, that proceeding from incifion is much the greateft, while the generated difeafe does not rife higher in the one cafe than in the other. So much for Cow-pox.

In a communication, which fome weeks ago I did myfelf the honour to fend you, I hinted my defign of inveftigating plague. I am at prefent, for this purpofe, foliciting admiffion,

ſion into the Peſt Hoſpital, ſupported by the contributions of the Franks at Smyrna; but its manager, a reverend friar, named Luigi, for reaſons inſcrutable to every one, perſiſts in what ſeems to me a narrow-minded, cruel, and unchriſtian denial. Should I find no ſop for this Cerberus, I will go from him to his conſtituents. I cannot believe they will be equally illiberal; but if in this reſpect I am likewiſe diſappointed, I ſhall endeavour to hold them up to that univerſal reprobation they will in ſuch caſe ſo eminently deſerve. In the mean time, I continue to believe that the plague is in no inſtance either a contagious or incurable diſeaſe. If I ſucceed in eſtabliſhing thoſe propoſitions, the world will, I truſt, rejoice in my ſucceſs. If I fail, the matter will be no worſe than at preſent. An enquiry, conducted in the manner I propoſe, will in all probability elicit ſome truths, which, for want of obſervers only, remain hitherto concealed.

To conclude, I pledge myſelf to two things; firſt, to make a near and perſonal obſervation of plague, in defiance of every obſtacle that may occur; and in the ſecond, if I ſurvive the enquiry, to make an accurate and faithful report of every obſerved ſymptom, and of the effect of every preſcribed remedy. However much attached to my preſent opinions, I ſhall haſten to recant them, the moment I diſcover them erroneous. All I aſk of the public, is to hear me with patience, and to judge of me with candour.

Entertaining ſentiments of reſpect for your valuable Publication, I have the honour to be, &c.

